# Surface Modifications of Superparamagnetic Iron Oxide Nanoparticles with Chitosan, Polyethylene Glycol, Polyvinyl Alcohol, and Polyvinylpyrrolidone as Methylene Blue Adsorbent Beads

**DOI:** 10.3390/polym16131839

**Published:** 2024-06-27

**Authors:** Linh Doan, Tam T. T. Nguyen, Khoa Tran, Khanh G. Huynh

**Affiliations:** 1 Department of Chemical Engineering, International University—Vietnam National University, Ho Chi Minh City 70000, Vietnam; 2 Nanomaterials Engineering Research & Development (NERD) Laboratory, International University—Vietnam National University, Ho Chi Minh City 70000, Vietnam; 3 School of Chemical and Environmental Engineering, International University—Vietnam National University, Ho Chi Minh City 70000, Vietnam; 4 School of Biomedical Engineering, International University—Vietnam National University, Ho Chi Minh City 70000, Vietnam

**Keywords:** adsorption, desorption, kinetics, methylene blue, synthetic dye, wastewater treatment

## Abstract

Due to the negative impacts the dye may have on aquatic habitats and human health, it is often found in industrial effluent and poses a threat to public health. Hence, to solve this problem, this study developed magnetic adsorbents that can remove synthetic dyes like methylene blue. The adsorbent, in the form of beads, consists of a polymer blend of chitosan, polyethylene glycol, polyvinyl alcohol, polyvinylpyrrolidone, and superparamagnetic iron oxide nanoparticles (average size of 19.03 ± 4.25 nm). The adsorption and desorption of MB from beads were carried out at pH values of 7 and 3.85, respectively. At a concentration of 9 mg/L, the loading capacity and the loading amount of MB after 5 days peaked at 29.75 ± 1.53% and 297.48 ± 15.34 mg/g, respectively. Meanwhile, the entrapment efficiency of MB reached 29.42 ± 2.19% at a concentration of 8 mg/L. The cumulative desorption capacity of the adsorbent after 13 days was at its maximum at 7.72 ± 0.5%. The adsorption and desorption kinetics were evaluated.

## 1. Introduction

Water is an important natural resource in the world, and ensuring its efficient management is crucial for safeguarding its future sustainability for both the environment and human survival [1]. Over the years, significant contamination has been noted, and certain technologies employed for remediation may generate secondary contaminants or byproducts, which can worsen environmental pollution [2]. Contaminants in wastewater originate from two main sources. The initial reason is natural processes like volcanic activity, soil erosion, and rock weathering, and the second source is human activities such as waste disposal, urban runoff, mining, printed circuit board manufacturing, agriculture, metal surface treatment, fuel combustion, textile dyeing, semiconductor production, and others [3,4].

To protect the diverse range of plant and animal life it supports, it is essential to implement measures that prevent contamination from both organic and inorganic pollutants [5]. Over time, there has been a consistent observation of severe contamination, with dyes often being cited among the persistent organic and mineral pollutants reported [6]. Manufactured on a global scale in significant quantities and diverse forms [7], dyes are categorized according to the source of their materials (natural or synthetic) and the nature of their chromophore or autochrome groups [6]. This classification contributes to their potential to enter water bodies, where they can hinder light penetration, resulting in a substantial detrimental effect on ecosystems by reducing photosynthetic activity.

Additionally, dyes pose severe risks as they are highly harmful and carcinogenic. Their accumulation in certain aquatic organisms presents a notable environmental threat, alongside the potential for adverse effects on human health, such as skin irritation, allergic dermatitis, cancer, and genetic mutations [8]. In industries, a significant volume of vibrant wastewater is generated, often containing toxins, resistant to biodegradation, and posing environmental sustainability challenges [9]. Several examples of artificial coloring agents encompass aniline blue, alcian blue, basic fuchsin, methylene blue (MB), crystal violet, toluidine blue, and congo red [10]. MB is among the harmful dyes utilized across different industries.

Methylene blue (MB) is among the harmful dyes utilized across different industries. MB, with the chemical formula C_16_H_18_N_3_SCl [11], is an aromatic heterocyclic basic dye. It is alternatively known as a cationic or primary thiazine dye. The presence of negative polar sites on water molecules results in an electrostatic attraction towards the cationic dye, causing the positive ions to separate and form a stable solution with water at ambient temperature [11].

MB has diverse applications across various industries, notably in textiles, where it serves as a dye for cotton, wool, and silk [12]. Additionally, MB is primarily used in the textile industry to impart a vibrant blue color to garments. Its chemical properties allow it to adhere effectively to the interstitial gaps of cotton fibers, ensuring that the dye remains stable and durable on the fabric [11]. This strong adherence and stability make MB one of the most popular and frequently used dyes in apparel manufacturing. Its widespread application is due to its ability to produce a consistent and long-lasting color, making it a staple choice for dyeing various types of clothing.

Since 7 × 10^7^ tons of synthetic dyes are dumped into the environment annually, numerous methods have been attempted across various technologies in wastewater treatment to ensure the safety of water supplies for consumption [13]. These methods include coagulation [14], electrochemical processes [15], biological treatment [16], adsorption [17], and photocatalytic activity [18]. Nonetheless, water treatment technologies demand extended treatment durations and incur substantial operational expenses. Adsorption seems to stand out as one of the most prevalent methods for eliminating MB [11]. The process of adsorption is recognized for its efficacy in treatment owing to its operational simplicity, cost-efficiency, versatility, and responsiveness towards harmful contaminants. Numerous adsorbents have been utilized for eliminating various categories of dyes. Among the frequently employed adsorbents are activated carbons, plant or lignocellulosic residues, clays, and biopolymers [19,20].

Moreover, nano-adsorbents prove to be a remarkably efficient technology for removing organic dyes from both water and wastewater. Their reduced size and expanded adsorptive surface area significantly enhance their effectiveness in this application. Some researchers have enhanced the adsorption capacity of materials by modifying them through combinations with other chemicals and substances. Examples of nano-adsorbents such as nanofibers, graphene, metal oxides, and carbon nanotubes have the potential to enhance water and wastewater treatment processes [21]. In the field of water treatment, the inclusion of additional nanoparticles (NPs) may facilitate the process of separation during the elimination of artificial dyes from water owing to their magnetic properties [22]. As a result, superparamagnetic iron oxide nanoparticles (SPIONs), also known as Fe_3_O_4_, are among the most frequently utilized materials.

Superparamagnetic NPs can find applications in diverse fields through surface modifications with various materials. In order to prevent aggregation and maintain the stability of the nanoparticles, SPIONs can be coated with organic compounds such as acids, polysaccharides, or polymers. However, various studies have shown that certain undesirable aggregates of SPIONs may exhibit reduced stability, biocompatibility, and effectiveness. Consequently, there has been research into combining SPIONs with other stabilizing agents, such as carbon compounds [23,24,25]. Therefore, modifying SPIONs with polymers is essential to improve their adsorption capabilities for MB, a synthetic textile dye that is both toxic and carcinogenic. The incorporation of polymers onto the surface of SPIONs can significantly enhance their interaction with MB molecules, thereby increasing the efficiency of the adsorption process. This modification is vital for the development of advanced materials aimed at effectively removing MB from wastewater, thereby reducing its harmful environmental and health impacts.

Although SPION nanoparticles possess a significant surface area, incorporating carbon-based materials like activated charcoal (AC), graphene oxide, and carbon nanotubes can substantially enhance the adsorption capacity of synthetic dyes [21,22,23]. This improvement is due to the larger surface area, superior adsorption properties, and favorable chemical structure of these carbon-based materials. Additionally, hydrophobic SPIONs can be produced through various methods, including co-precipitation, hydrothermal, and the sol-gel approach. Among these, co-precipitation stands out as the quickest method because of its controlled size and magnetic properties, yielding the highest amount of magnetite and offering an easy synthesis process by minimizing maghemite formation, despite some drawbacks such as particle agglomeration [26]. SPIONs enhance the separation process due to their magnetic properties and offer a considerable surface area.

To avoid aggregation, SPIONs can be modified using polymers. Recently, chitosan was used to modify SPION. Chitosan (CS) is a cationic polyelectrolyte prepared by N-deacetylation of chitin, also named poly (β-1-4)-2-amino-2-deoxy-D-glucopyranose [27]. CS exhibits non-toxic, hydrophilic, biocompatible, biodegradable, and antibacterial properties. These characteristics have spurred its versatile application across various sectors, including biomedicine, cosmetics, food, and textiles [28]. CS has been identified as an appropriate natural polymer for the adsorption of metal ions [29], as the amino (–NH_2_) and hydroxyl (–OH) groups present on the chitosan chain can function as sites for chelating metal ions. Nevertheless, the behavior of CS is greatly influenced by the pH level, leading to its ability to transition between a gel state and a dissolved state based on the pH values [19]. Hence, CS has shown promise as an adsorbent in wastewater treatment.

Multiple research studies have focused on altering the surface of chitosan (CS) by chemical means, either through uniform or varied crosslinking processes involving di- or polyfunctional substances. This has been aimed at enhancing mechanical properties, adsorption capacity, or preventing the dissolution of chitosan in acidic environments. Li et al. developed a material composed of CS-coated magnetic mesoporous silica NPs and applied it effectively for the removal of MB from water [30]. Furthermore, Hoa et al. published a paper focusing on the adsorption characteristics of porous beads made of hydroxyapatite/graphene oxide/chitosan toward MB [31]. In addition, the adsorption capacity of SPION-based MB adsorbents can also be influenced by the presence of CS. According to some studies, the adsorption capabilities of SPION/CS/graphene oxide and SPION/PVA/CS/graphene oxide may be 30.01 and 36.4 mg/g, respectively [25,32]. Thus, the significant effectiveness of combining CS with SPIONs holds promise for wastewater treatment.

Furthermore, polyethylene glycol (PEG), also referred to as H(OCH_2_CH_2_)_n_OH, is a synthetic polymer consisting of repetitive ethylene glycol units. When dissolved, each ethylene glycol unit binds to approximately two water molecules, resulting in a molecular size that is 5–10 times greater than that of proteins or other macromolecules of comparable molecular weights [33,34].

PEG finds widespread application in tissue engineering, drug delivery, electronics, and fluorescence detection owing to its antimicrobial characteristics and non-toxic nature [35]. PEG is a widely used agent for enhancing the biocompatibility of material surfaces that interact with cells. The modification of polyvinyl chloride (PVC) resin with PEG enhanced its blood compatibility [36]. Furthermore, PEG has the capability to serve as active sites by virtue of the polyethylene oxide chains’ capacity to create durable complexes with metal cations resembling crown ethers. PEG is appealing for chemical synthesis due to its ease of functionalization with various groups such as azides, thiols, carboxylic acids, hydroxyls, and epoxides [37]. In order to uphold electroneutrality, it is necessary for these complexes of PEG-metal cations to incorporate a corresponding anion into the organic phase. This ensures that the anion is present for potential interactions with the organic reactants [38]. Due to its high solubility in water and non-absorption by the human body, PEG is frequently used in various medical applications. It is utilized in anticancer drugs, organ preservation, and tablet formulation, where it functions as a lubricant and binder [35]. PEG’s higher viscosity compared to other meltable binders makes it suitable for achieving melt agglomeration through the immersion mechanism. Some PEGs are available as solid beads. For example, melt agglomeration studies can utilize PEG beads of various sizes to investigate the mechanisms behind the formation and growth of agglomerates under these processing conditions [39].

Moreover, PEG, an affordable synthetic non-ionic polymer, enhances the pore volume and dispersion of the composite material. When incorporated with CS, this organic porogen selectively dissolves within the polymeric matrix, generating macroporous networks [40].

On the other hand, polyvinyl alcohol (PVA) is a semi-crystalline or linear synthetic polymer that appears creamy or whitish with a tasteless, odorless, non-toxic, and thermostable nature, typically found in granular or powdered form [41]. It is a derivative of a vinyl polymer linked solely by C–C bonds [42].

PVA possesses excellent formability and is extensively utilized as a carrier to produce PVA composites with specific mechanical strength [43]. PVA is commercially available in various grades, distinguished by viscosity and degree of hydrolysis [42]. Moreover, PVA exhibits excellent solubility in water because of the abundance of hydroxyl groups in its molecular structure and can undergo biodegradation within a relatively brief period. PVA offers additional benefits such as high biocompatibility, hydrophilicity, and the capacity to form fibers capable of retaining significant quantities of water and/or biological solutions, all while preserving their structural integrity under deformation [44]. Polyvinyl alcohol (PVA) is a synthetic polymer characterized by its semi-crystalline or linear structure, which presents a creamy or whitish appearance. It possesses the attributes of being tasteless, odorless, non-toxic, and exhibiting thermostable properties. This polymer is commonly encountered in either granular or powdered form [42]. For both the strength and formation of beads, a pH range of 4–6 is necessary. However, a higher pH level is preferable for enhancing the strength and stability of PVA beads.

Several publications have demonstrated the use of PVA in wastewater treatment for the removal of toxic dyes in different forms. PVA beads and CS/PVA hydrogel beads have been extensively studied as efficient adsorbents for heavy metal ions and dyes [45]. Jeong et al. conducted a study assessing the effectiveness of a water purification system composed of PVA gel beads incorporating photosynthetic bacteria [46]. In pursuit of the study’s objectives, PVA surfaces were employed for the adsorption of MB.

Moreover, polyvinylpyrrolidone (PVP, also known as povidone or polyvidone) is denoted by the molecular formula (C_6_H_9_N_O_)_n_, derived from its monomer N-vinylpyrrolidone. It is a bulky, linear homopolymer, a non-toxic, non-ionic polymer containing functional groups such as C=O, C-N, and CH_2_ [47].

It is accessible in different molecular weights, and it exhibits high solubility in both water and various organic solvents, commonly employed in NP synthesis. These attributes, coupled with PVP’s capacity to form complexes with polar molecules and its biodegradability, have resulted in its widespread industrial usage, notably in pharmaceuticals and processed foods [37]. PVP is a preferred alternative for medication delivery systems due to its biocompatibility.

In addition to being a hydrophilic polymer, PVP exhibits great solubility in solvents with varied polarity, good binding capabilities, and a stabilizing impact for suspensions and emulsions. PVP is considered to be physiologically suitable for both animal and human use. Its utilization as a blood plasma extender, carrier for drugs, suspending agent (specifically as a protective colloid), and aid for tableting has been observed in both the United States and Europe [48]. The solubility of PVP in water and various non-aqueous solvents is attributed to the presence of the highly polar amide group located within the pyrrolidone ring, along with the apolar methylene and methine groups found in the ring and along its backbone [49]. PVP may create hydrogen bonds with CS, functioning as a cross-linker, which improves its mechanical characteristics. The presence of PVP improves the thermal stability of CS.

Hence, in response to this challenge, the aim of the study on surface modifications of SPIONs with chitosan (CS), polyethylene glycol (PEG), polyvinyl alcohol (PVA), and polyvinylpyrrolidone (PVP) as MB adsorbent beads is to develop novel materials for removing this hazardous dye from aqueous solutions. Additionally, this study can be used as the foundation to further develop dual-function materials with applications in dye removal and antimicrobial agents since the polymer blend that was used in this research exhibited antimicrobial activity [50].

## 2. Materials and Methods

### 2.1. Materials

Sodium hydroxide (NaOH), polyethylene glycol—1000 (PEG), iron (II) chloride tetrahydrate (FeCl_2_·4H_2_O), iron (III) chloride hexahydrate (FeCl_3_·6H_2_O), hydrochloric acid (HCl), and ammonia solution (NH_4_OH) were purchased from Xilong Scientific Co., Ltd. (Shantou, China). Chitosan (CS), polyvinylpyrrolidone (PVP K30) from Shanghai Zhanyun Chemical Co., Ltd. (Shanghai, China). Glacial acetic acid (AA) was purchased from RCI Labscan (Bangkok, Thailand). Polyvinyl alcohol (PVA) was purchased from Wuxi Yatai United Chemical Co., Ltd. (Shantou, China) All chemicals were used as received.

### 2.2. Methods

#### 2.2.1. Synthesis of SPION and M8C

SPION was synthesized individually, similar to previous publications, without any modifications [51]. The synthesis of the polymer blend M8C followed a procedure outlined in a previous study by Linh et al., with specific modifications as detailed [50]. Specifically, the M8C variant was prepared by preparing PEG, PVA, and PVP individually by adding the polymers with DI under constant stirring and heating at 80 °C. These polymers were synthesized by adding 2 g of each polymer to 50 mL of DI. Additionally, the CS mixture was synthesized by mixing 1 g of CS in 70 mL of a 3% AA solution under constant stirring and heating at 80 °C. Then, these polymers were blended together under constant stirring and heating at 80 °C.

#### 2.2.2. Synthesis of SPION/M8C Composite

The SPION/M8C composite was initially synthesized by mixing 30 mL of the M8C blend with 0.5 g of SPION. Then, the mixture was sonicated for 30 min. Then, the mixture was carefully added dropwise to a 9 M NaOH solution, allowing for controlled incorporation. Subsequently, the beads were extensively washed with distilled water (DW) until the pH of the filtrate reached neutrality. After washing, the beads were transferred to a glass dish and left to air dry overnight in an oven set at 80 °C.

#### 2.2.3. MB Adsorption

Multiple experiments were conducted using MB solutions at various concentrations to establish the calibration curve for adsorption. Following this, the concentration of nanoparticles was measured at room temperature over a specific duration. Specifically, 25 mL of MB solution at concentrations of 8 mg/L, 9 mg/L, and 10 mg/L, all adjusted to a pH of 7.0, were combined with 0.2 g of the composite beads in a 50 mL falcon tube. Subsequently, the tube was left to stand at room temperature for a period of 5 days.

Samples—aliquots—were collected and transferred into the cuvette using plastic pipettes and a neodymium magnet every 120 h to ensure enough observation intervals and analyzed using UV–Vis spectroscopy (Hach DR6000, Loveland, CO, USA). After measuring the absorbance, the aliquots were transferred back into the falcon tubes. To maintain data integrity and improve result accuracy, this adsorption experiment was carefully performed three times, ensuring consistency and dependability in the collected data.

#### 2.2.4. MB Desorption

Following the end of the adsorption experiment, neodymium magnets were used to remove an aliquot from each falcon tube, ensuring effective separation of the loaded adsorbents. Subsequently, a solution with a pH of 3.85 was prepared by combining DW with 2 M HCl. Each falcon tube was then filled with 25 mL of this acidic solution to initiate the desorption experiment at room temperature.

During the desorption process, the loaded adsorbents were allowed to release the MB for 13 days while maintaining a pH of 3.85 throughout the experiment. Following the required desorption interval, the samples were analyzed using UV–Vis spectroscopy to determine the amount of MB released. After analysis, the aliquots were reintroduced into their respective falcon tubes for further testing. To confirm the accuracy and uniformity of the data, this desorption operation was rigorously performed three times.

#### 2.2.5. Calculation

As shown in Equations (1)–(19), the loading amount (Q_t_), loading capacity (%LC), entrapment efficiency (%EE), adsorption kinetics models (pseudo 1st order—PFO, pseudo 2nd order—PSO), intraparticle diffusion, Elovich kinetic model, and desorption models (0th order, Higuchi, and Korsmeyer–Peppas) were used to fit the experimental data similar to previous publications [25,51].

(1) $$Loading\;amount\;\;Q_{t}=\frac{\left(C_{0}-C_{t}\right)V}{m}$$

(2) $$Loading\;capacity\;\%\mathrm{LC}=\frac{Weight\;of\;MB\;adsorbed\;on\;to\;the\;particles\;(mg)}{Weight\;of\;particles\;\left(mg\right)}\times 100$$

(3) $$Entrapment\;Efficiency\;\%EE=100\times \;\frac{Weight\;of\;MB\;adsorbed\;on\;to\;the\;\;particles\;(mg)}{Weight\;of\;MB\;initially\;fed\;(mg)}$$

(4) $$PFO\;nonlinear\;Q_{t}=Q_{e}(1-e^{-kt})$$

(5) $$PFO\;linear\;\log\left(Q_{e}-Q_{t}\right)=\log Q_{e}-\left(\frac{k_{1}}{2.303}\right)t$$

(6) $$PSO\;nonlinear\;Q_{t}=Q_{e}(1-e^{-kt})$$

(7) $$\;PSO\;linear\;type\;I\;Q_{t}=\frac{k_{2}Q_{e}^{2}t}{1+k_{2}Q_{e}t}$$

(8) $$PSO\;linear\;type\;II\;\frac{1}{Q_{t}}=\frac{1}{k_{2}Q_{e}^{2}}+t/Q_{e}$$

(9) $$PSO\;linear\;type\;III\;\frac{1}{Q_{t}}=\left(\frac{1}{k_{2}Q_{e}^{2}}\right)\frac{1}{t}+\frac{1}{Q_{e}}$$

(10) $$PSO\;linear\;type\;IV\;Q_{t}=Q_{e}-\left(\frac{1}{k_{2}Q_{e}}\right)\frac{Q_{t}}{t}$$

(11) $$Zeroth\text{-}order\;linear\;M_{t}=k_{0}t$$

(12) $$Zeroth\text{-}order\;nonlinear\;M_{t}=k_{0}t$$

(13) $$Korsmeyer–Peppas\;linear\;\log\left(\frac{M_{t}}{M_{\infty }}\right)=\log\left(k_{KP}\right)+n_{KP}\log\left(t\right)$$

(14) $$Korsmeyer–Peppas\;nonlinear\;\frac{M_{t}}{M_{\infty }}=(k_{KP})(t^{n_{KP}})$$

(15) $$Higuchi\;linear\;\log\left(M_{t}\right)=\log\left(k_{H}\right)+0.5\log\left(t\right)$$

(16) $$Higuchi\;nonlinear\;M_{t}=k_{H}t^{1/2}$$

(17) $$Intraparticle\;diffusion\;Q_{t}=I+{k_{i}t}^{1/2}$$

(18) $$Simplified\;Elovich\;model\;Q_{t}=\beta \;\ln\left(\alpha \beta \right)+\beta \ln t$$

(19) $$Chi\text{-}square\;test\;\chi^{2}=\sum_{i=1}^{m}\frac{{\left(Q_{t,exp}-Q_{t,calc}\right)}^{2}}{Q_{t,calc}}$$

#### 2.2.6. Characterization

The use of field emission scanning electron microscopy (FE-SEM, Hitachi, SU8000, Tokyo, Japan), Fourier transform infrared spectroscopy (FTIR, Tensor 27, Bruker, MA, USA) was important to confirm the successful construction of the SPION/M8C beads material. The FE-SEM provided detailed images revealing the material’s morphology, while FTIR identified the iron oxide nanoparticle formation. XRD analysis confirmed the characteristics of the iron oxide nanoparticles, demonstrating their superparamagnetic nature. A vibrating sample magnetometer (VSM, Tensor 27, Bruker, Germany) may be used to confirm the existence of superparamagnetism in iron nanoparticles. Moreover, techniques such as Brunauer–Emmett–Teller (BET) or Barrett–Joyner–Halenda (BJH) can assess the surface area, pore size, and volume. In addition, the swelling ratio of the beads can be determined and calculated using the following equation [52]:

(20) $$Swelling\;ratio=\;\frac{w_{wet}-w_{dry}}{w_{dry}}$$

where w_wet_ is the mass of wet beads and w_dry_ is the mass of dry beads.

## 3. Results and Discussion

### 3.1. FE-SEM Analysis

**Figure 1:**
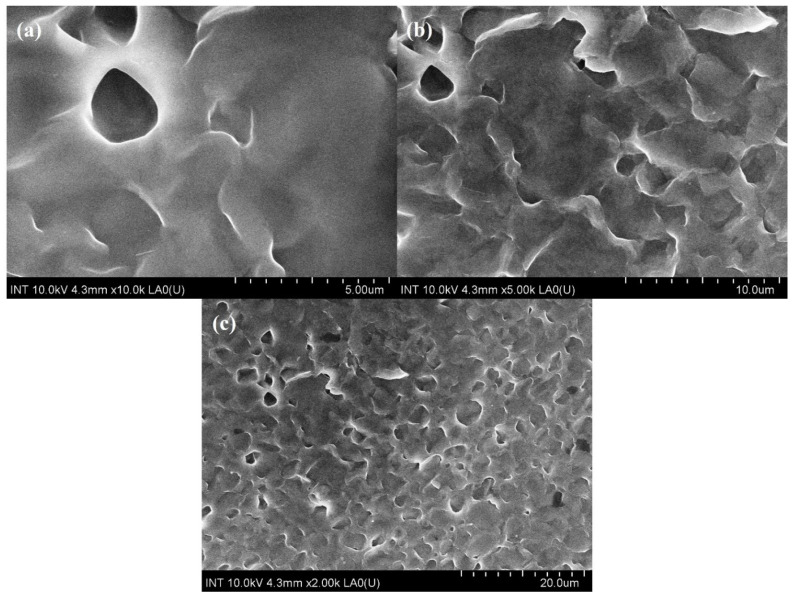
FE-SEM images of M8C at (**a**) 5 μm, (**b**) 10 μm, and (**c**) 20 μm.

Scanning electron microscopes (SEM) are extensively utilized for the study and analysis of microparticle and nanoparticle imaging and the characterization of solid materials. A specific type of SEM, known as a field emission scanning electron microscope (FE-SEM), uses negatively charged electrons emitted from a field emission source instead of light for imaging. Figure 1 represents M8C’s (polymer blend of PVA, PVP, PEG, and CS) morphology.

It is clearly seen that the combination of several polymer components with varying characteristics would likely give M8C’s surface an uneven or rough appearance, based on Figure 1. Pores or other visible characteristics may be present, depending on the circumstances and manner of production.

M8C had some porosity, depending on composition and processing technique. The creation of porous structures during the drying or crosslinking processes, the existence of empty spaces between polymer particles, or trapped gas bubbles during synthesis might all contribute to this porosity.

**Figure 2:**
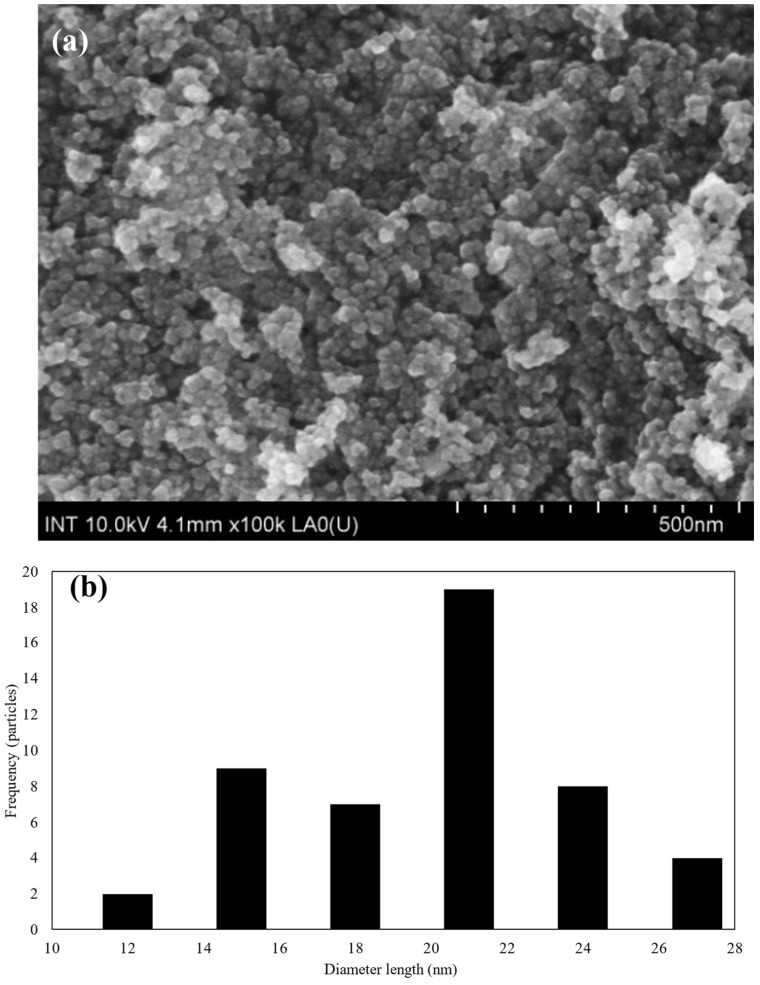
(**a**) The morphology of SPIONs at 500 nm and (**b**) the normal size distribution of SPIONs.

On the other hand, the morphology of the superparamagnetic iron oxide nanoparticles (SPIONs) is illustrated in Figure 2.

Based on the FE-SEM images, the morphology, as shown in Figure 2a, the size distribution, as shown in Figure 2b, and the average size of SPIONs were estimated. The results were 19.03 ± 4.23 nm, which is in line with previous publications [53]. When zooming into higher magnifications, individual SPIONs came into focus, revealing their spherical or quasi-spherical morphology. Additionally, the size distribution is measured in terms of nanometers (nm), with a range of 10 to 30 nm. It can be seen from this distribution that the majority of particles have sizes between 18 and 20 nm.

**Figure 3:**
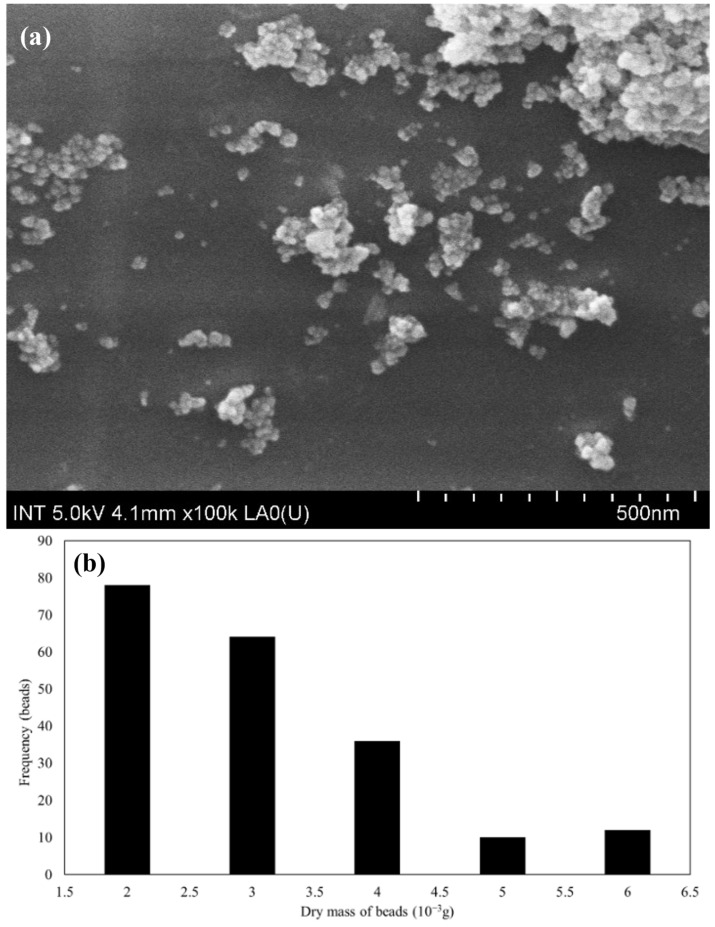
(**a**) FESEM of SPION/M8C beads at 500 nm and (**b**) the normal mass distribution of SPIONs.

In addition, Figure 3 depicts the morphology of SPION/M8C beads.

Using ImageJ software (v.1.53t) to analyze the FE-SEM images, the average size was found to be 22.32 ± 4.44 nm, which is displayed in Figure 3a. The illustrations showed that the composite structure was heterogeneous, with various polymer components and SPIONs dispersed throughout the beads. It demonstrated an uneven distribution of SPIONs in the polymer. Furthermore, observable surface protrusions or abnormalities might mean that SPIONs or polymer aggregates were present on the bead surface.

This distribution analysis in Figure 3b revealed that the majority of the synthesized beads had a mass ranging between 0.002 and 0.004 g after undergoing the drying process. These beads were presented in significant quantities, indicating a relatively uniform synthesis process. However, a smaller subset of the beads exhibited a mass between 0.005 and 0.006 g. The variation in bead mass can be attributed to the dissolution and distribution behavior of the SPIONs during the synthesis. During the sonication process involving the M8C polymer blend and SPIONs, the typically insoluble SPION particles became well-dispersed throughout the mixture. This effective dispersion ensured that each droplet of the mixture, when introduced into the NaOH solution, incorporated a consistent amount of SPIONs, thereby increasing its mass.

### 3.2. FTIR Analysis

**Figure 4:**
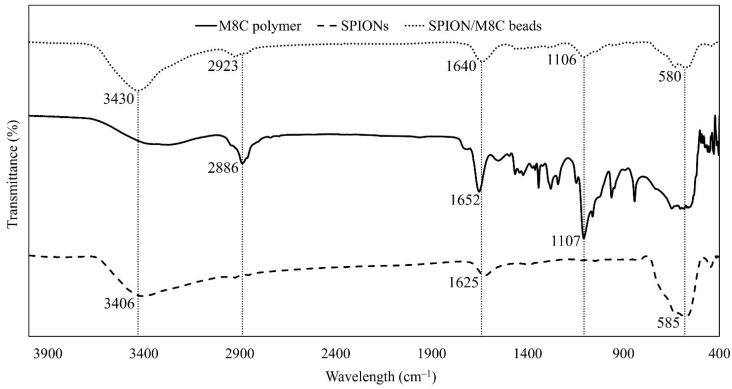
FTIR spectrum of M8C, SPIONs, and SPION/M8C beads.

FTIR proves to be a useful tool for quantifying and identifying the functional groups within nanoparticles, thus confirming the material’s final structure. Following the synthesis of SPION/M8C beads, the sample underwent FTIR analysis, as shown in Figure 4.

As illustrated in Figure 4, the peaks of SPION/M8C beads underwent analysis. The intensity of O–H symmetric stretching stood out, likely due to the low concentration of pure polymers in water. However, the characteristic peaks of each component could be distinctly identified, including the vibrations of Fe–O in SPIONs and SPION/M8C beads, the Amide I band, CH_2_ asymmetric stretching vibration, and the N–H groups of CS + AA. Based on Figure 4, the FTIR spectrum was further analyzed and shown in Table 1.

As shown in Table 1, upon analysis of the FTIR spectrum of M8C, it was evident that there were four distinct peaks observed at 2886 cm^−1^, 1652 cm^−1^, and 1107 cm^−1^. These peaks were associated with specific molecular vibrations, specifically an asymmetric C–H stretching vibration [57], C=O stretching vibration, and C–O–C symmetric stretching.

The vibrational spectra of SPIONs exhibited the emergence of three peaks. The first peak, at 3406 cm^−1^, was attributed to O–H stretching, while the carbonyl C=O stretching band and the vibration of Fe–O were observed at 1625 cm^−1^ and 585 cm^−1^, respectively.

In the FTIR spectrum for the composite of SPION/M8C beads, the observed peaks closely align with those of the individual components, though some slight shifts are present. It is noteworthy that the peak observed at 3430 cm^−1^ likely corresponds to the O–H stretching present in all components, whereas the peak at 2923 cm^−1^ may be associated with the asymmetric C–H stretching vibration. Additionally, the peak at 1640 cm^−1^ corresponded to the carbonyl stretching vibration. Moreover, the absorption band observed at 1106 cm^−1^ likely corresponds to the free amino group located at the glucosamine C2 position of CS, or it may be indicative of C–O–C stretching.

Additionally, the characteristic vibration of Fe–O in SPIONs is also evident in the spectrum of the nanocomposites, with peaks appearing at both 583 cm^−1^ and 580 cm^−1^. These features further highlight the presence and interaction of the various functional groups within the composite material. Due to the presence of surfactant on the particles’ surface, the peaks may have shifted by a few cm^−1^ compared to the compositional peaks in the FTIR spectra of other samples. Consequently, this FTIR analysis confirmed that the IONPs were indeed SPIONs and that the compound was successfully synthesized.

### 3.3. XRD Analysis

**Figure 5:**
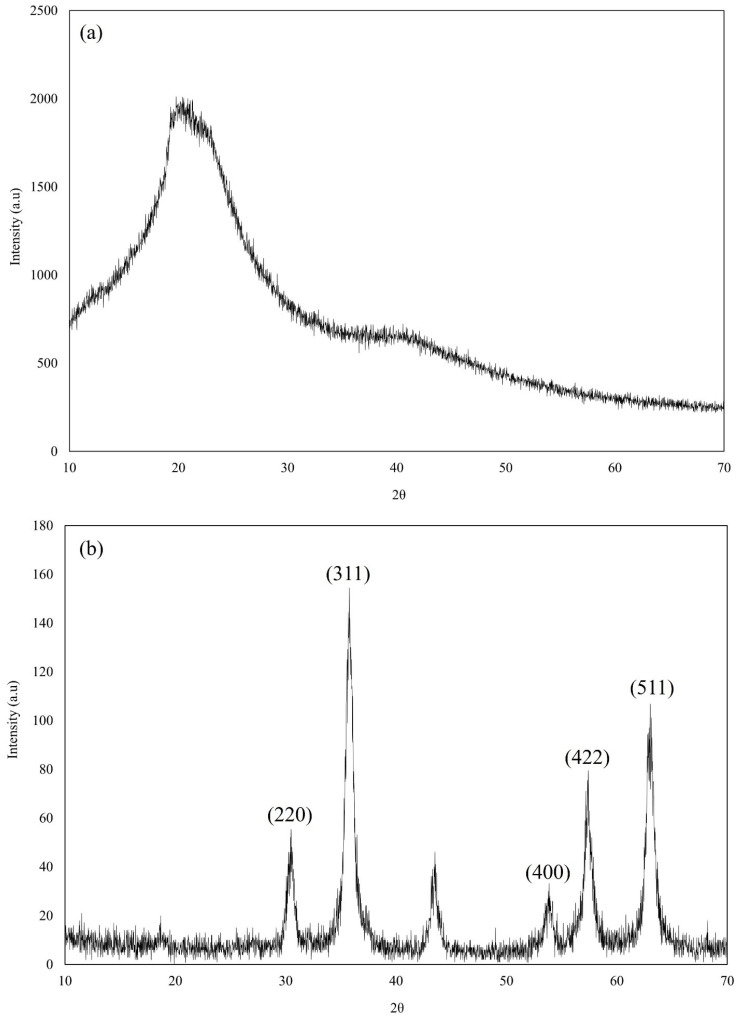

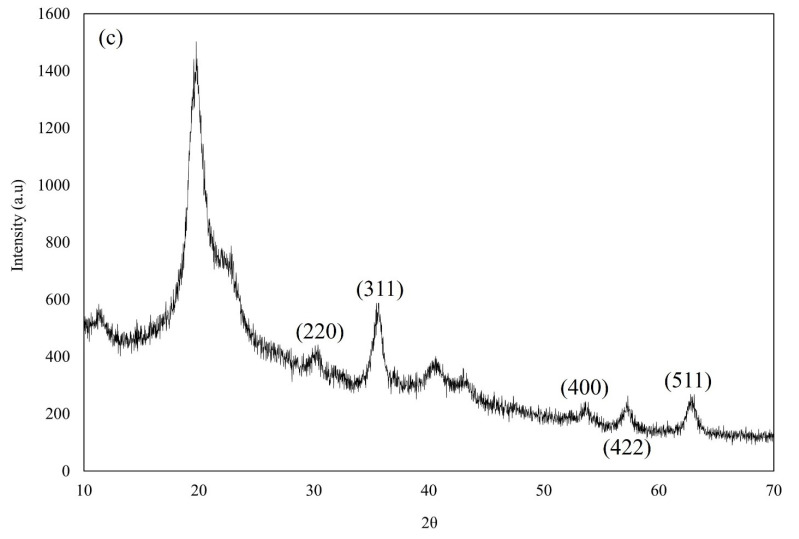
Overlay X-ray diffractogram of (**a**) M8C, (**b**) SPIONs, and (**c**) SPION/M8C beads.

XRD is a commonly used technique for studying material crystalline structure. It offers comprehensive details on a sample’s structural characteristics, crystallinity, and phase composition of SPIONs and SPION/M8C beads, as shown in Figure 5.

Figure 5b displays X-ray diffractograms showcasing distinct peaks located at specific angles such as 30.47°, 35.76°, 53.85°, 57.41°, and 63.01° [11]. These peaks, characteristic of magnetite, served as significant evidence indicating the nanoparticles’ composition as SPIONs. Similarly, in Figure 5c, the X-ray diffractograms of SPION/M8C beads exhibited notable peaks at angles of 30.05°, 35.53°, 53.60°, 57.18°, and 62.83° [11]. These pronounced peaks strongly suggest the presence of iron within the SPION/M8C bead composite, reinforcing its composition analysis.

The prominent peaks detected at angles of 30°, 35°, 54°, 57°, and 63° in the XRD pattern of SPIONs correspond precisely to the crystallographic planes (*hkl*) (220), (311), (400), (422), and (511) within the spinel cubic lattice structure characteristic of SPIONs [11]. This correlation strongly suggests that the magnetic core material (M8C) has been effectively adorned with SPIONs, demonstrating the successful decoration of the M8C with SPIONs. Combining the results of FE-SEM, FTIR, and XRD, further investigations should be conducted to determine whether the SPION was modified on the surface permanently.

### 3.4. VSM Analysis

**Figure 6:**
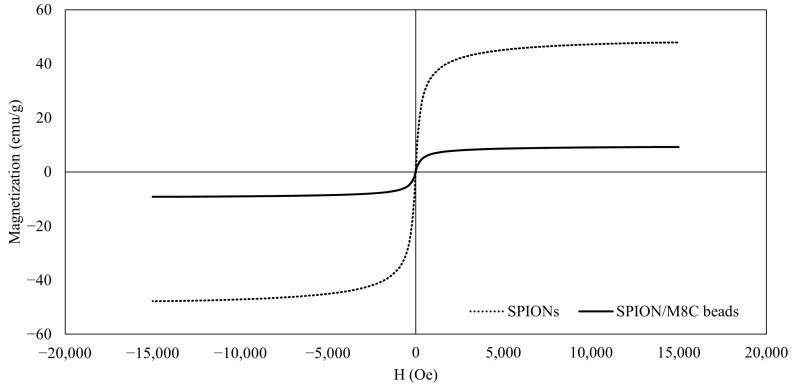
VSM results of SPIONs and SPION/M8C beads.

The VSM curves showed that the IONPs behaved in a superparamagnetic manner, as observed in Figure 6. In line with other assessments, this result confirms that the IONPs are superparamagnetic [25].

Similarly, even after the surface modification of SPIONs with CS, PEG, PVA, and PVP, the superparamagnetic properties remained intact. However, there were significant decreases in magnetization, likely due to the presence of the polymer shell coatings surrounding the nanoparticles.

The magnetic properties of both SPIONs and SPION/M8C beads were assessed using VSM at room temperature. Across all samples, the magnetization curves exhibited characteristic S-shaped profiles in response to the applied magnetic field, with no observable remanence or coercivity. These findings were indicative of superparamagnetic behavior across the board. Specifically, the magnetization curve depicted in Figure 6 for SPIONs showcases this superparamagnetic behavior. Furthermore, the saturation magnetization values, averaging around 51 emu/g, aligned closely with approximately 54% of the bulk magnetite content [58].

### 3.5. BET/BJH Analysis

**Figure 7:**
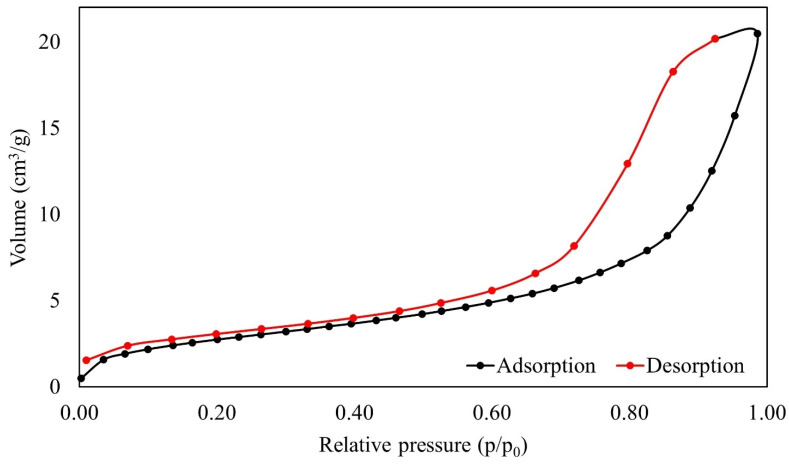
The BET results for relative pressure over the volume of SPION/M8C beads.

The volume of adsorbate uptake initiates at a relative pressure and gradually rises with higher relative pressures, as shown by the adsorption–desorption isotherm, as described in Figure 7. This pattern shows how the adsorbate and the adsorbent interact dynamically over a variety of pressures, increasing the material’s ability to retain the adsorbate as the relative pressure rises.

The adsorption/desorption isotherm analysis of SPION/M8C beads revealed a type IV isotherm, indicating adsorbents with wide pore size distributions [11,25]. This structural characterization suggests a complex pore architecture, which could contribute to enhanced adsorption properties and surface reactivity. It is characterized by pores ranging in size from 2 to 50 nm [25]. The average pore diameter of the synthesized SPION/M8C beads, determined using the BJH method, is summarized in Table 2.

The results obtained from the BJH and BET tests yield valuable insights into the surface characteristics of the material, as outlined in Table 2. Notably, it was discovered that the surface areas for desorption and adsorption were measured at 25.89 m^2^/g and 16.71 m^2^/g, respectively.

Moreover, examining the SPION/M8C beads sample revealed peak values for both total pore volume and pore diameter during adsorption, reaching 0.036 cm^3^/g and 9.82 Å, respectively. Similarly, the pore diameter and volume during desorption were determined to be 90.281 Å and 0.034 cm^3^/g, respectively. These findings collectively contribute to a comprehensive understanding of the material’s structural characteristics and their correlation with its adsorption capabilities.

### 3.6. Swelling Ratio Analysis

The swelling ratio (SR) of beads, including polymer and hydrogel beads, is determined similarly to other materials. It quantifies the extent to which the beads swell upon absorbing a liquid relative to their original dry state.

Cycle I beads had a wet weight of 0.0398 ± 0.005 g and a dry weight of 0.0032 ± 0.001 g. During cycle II, the wet weight reduced to 0.0243 ± 0.004 g, whereas the dry weight was 0.0029 ± 0.001 g. These findings revealed a significant drop in wet and dry weights between the two cycles.

**Figure 8:**
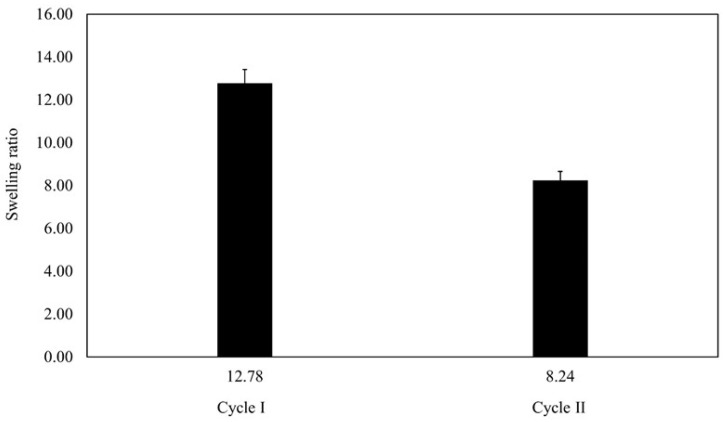
The average SR of cycle I and cycle II.

Figure 8 presents the average SR for each cycle. In cycle I, the average SR was 12.78 ± 4.48, whereas in cycle II, it dropped to 8.24 ± 3.14. These findings meant that the beads were capable of absorbing and retaining nearly 13 times their weight in the swelling medium throughout cycle I, demonstrating their remarkable absorbency. Thus, the comparison of the two cycles highlighted the dynamic nature of the beads’ swelling behavior as well as their capacity to efficiently absorb and hold moisture.

**Figure 9:**
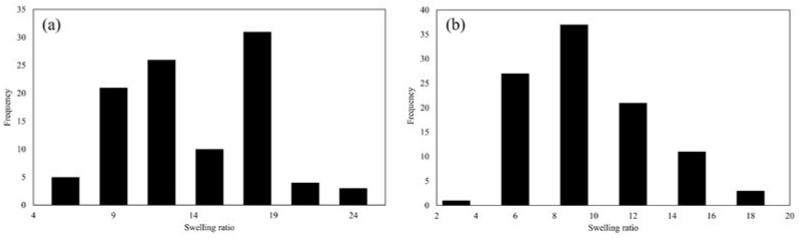
The swelling ratio of (**a**) cycle I and (**b**) cycle II.

Based on Figure 8, the average swelling ratio after the second cycle is lower than the first cycle. Specifically, the swelling ratio distribution of each cycle can be seen in Figure 9.

According to Figure 9, it was observed that during cycle I, the majority of beads displayed a swelling ratio spanning from 10 to 20. Beads with a swelling ratio of around 10 were found to absorb liquid approximately 10 times their dry weight or volume, signifying a moderate level of absorbency. Conversely, beads with a swelling ratio around 20 exhibited the ability to absorb liquid up to 20 times their dry weight or volume, indicating a notably higher absorbent capacity.

Furthermore, the size and form of the beads had a considerable impact on their swelling behavior. Smaller beads had a higher surface area-to-volume ratio, which improved their absorption capabilities. This indicated that smaller beads may have a more noticeable swelling propensity than bigger ones due to the greater surface area available for interaction with the liquid medium.

### 3.7. Adsorption

At hourly intervals, the loading amount (Q_t_), entrapment efficiency (%EE), and percent loading capacity (%LC) for the adsorption process were identified and presented in Table 3. These calculations were carried out using the UV–Vis spectrometry results for MB, which were obtained at a wavelength of 664 nm.

According to Table 3, the highest Q_t_ and %LC of MB at 120 h were observed at an initial MB concentration of 9 mg/L. Specifically, the Q_t_ reached 297.48 ± 15.34 mg/g, and the %LC was 29.75 ± 1.53%. At this concentration, the adsorbent demonstrated optimal performance in terms of the amount of MB it could adsorb and the efficiency of its capacity utilization.

On the other hand, the highest %EE was recorded at an initial MB concentration of 8 mg/L, with a value of 29.42 ± 2.19%. This indicated that, at this concentration, a significant proportion of MB was successfully entrapped within the adsorbent relative to the initial amount.

**Figure 10:**
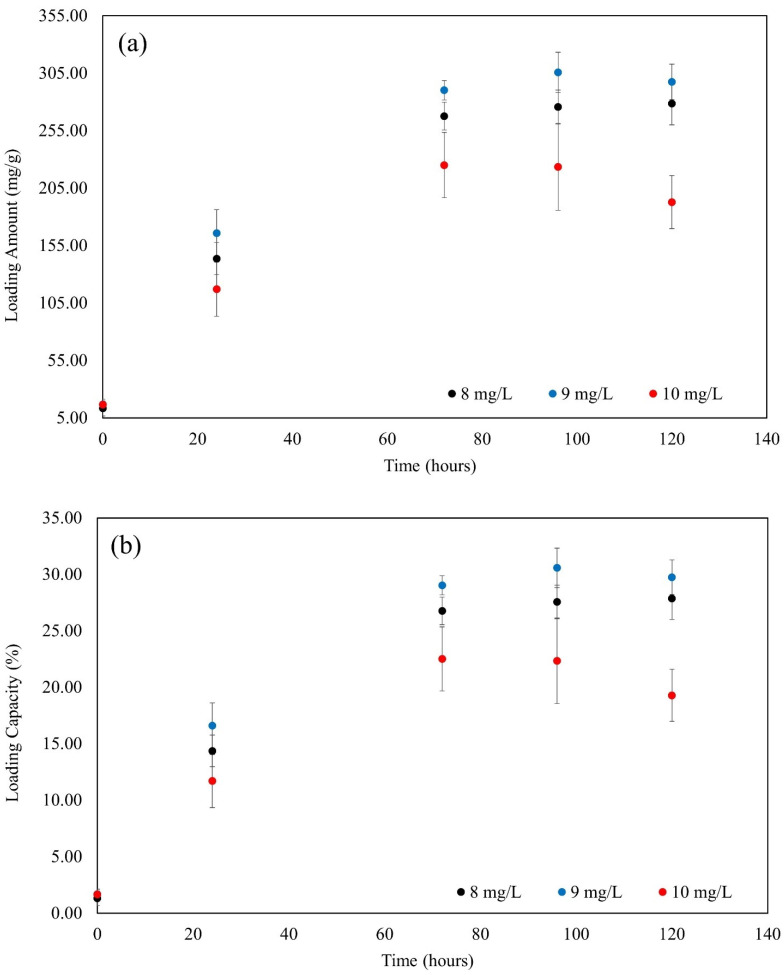

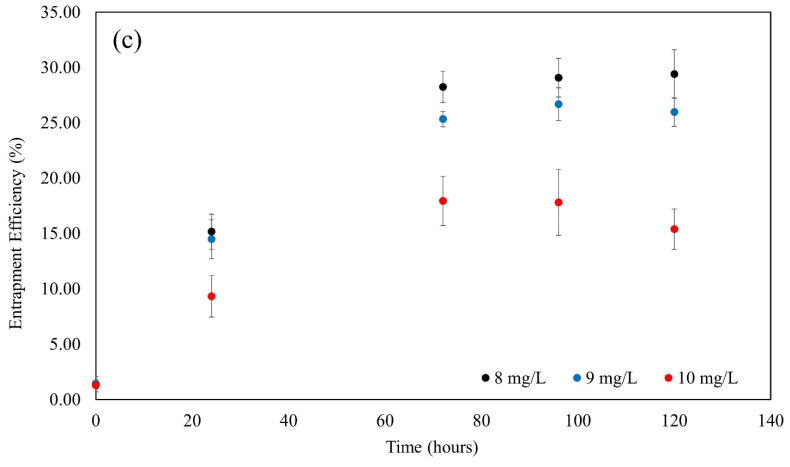
(**a**) The adsorption amount with time; (**b**) the %LC with time; and (**c**) the %EE with time.

Conversely, at an initial MB concentration of 10 mg/L, the values for Q_t_, %LC, and %EE were the lowest among the concentrations tested. The Q_t_ was 192.82 ± 23.18 mg/g, the %LC was 19.28 ± 2.32%, and the %EE was 15.39 ± 1.83%. These lower values suggested that, at higher MB concentrations, the adsorption efficiency and capacity utilization of the adsorbent decreased, possibly due to saturation effects or limitations in the adsorbent’s ability to handle higher concentrations effectively. As shown in Figure 10, the Q_t_, %LC, and %EE were evaluated over time.

After one day at room temperature, the amount of MB increased significantly in three different concentrations, as depicted in Figure 10a, roughly 100 mg/g. These percentages correspond to concentration increases of 8, 9, and 10 mg/L. It is evident from this that the adsorption capacity is influenced by concentration.

The patterns of the %LC of MB in Figure 10b and the quantity of MB in Figure 10a were identical. When comparing the Q_t_ and %LC results after 24 and 72 h, they were nearly twice as high. Moreover, it could be clearly seen that the starting concentration of 9 mg/L, Q_t,_ and %LC both achieved their maximum value and kept growing until they reached equilibrium at 10 mg/L.

As shown in Figure 10c, the concentration of 8 mg/L produced the greatest percentage for the %EE of MB after 24 h. The %EE had reached about 15% at this stage. Over time, this proportion grew steadily, and before the system achieved equilibrium at 120 h, the %EE sharply rose to 30%.

Nonetheless, it is evident that the Q_t_, %LC, and %EE of the 10 mg/L concentration consistently fall to the bottom. As can be seen, all three values (Q_t_, %LC, and %EE) rose daily but either held steady after 72 h or even slightly declined. This is because an absorbance of a concentration greater than 10 is outside the range, and a concentration of 10 mg/L is the maximum concentration.

**Figure 11:**
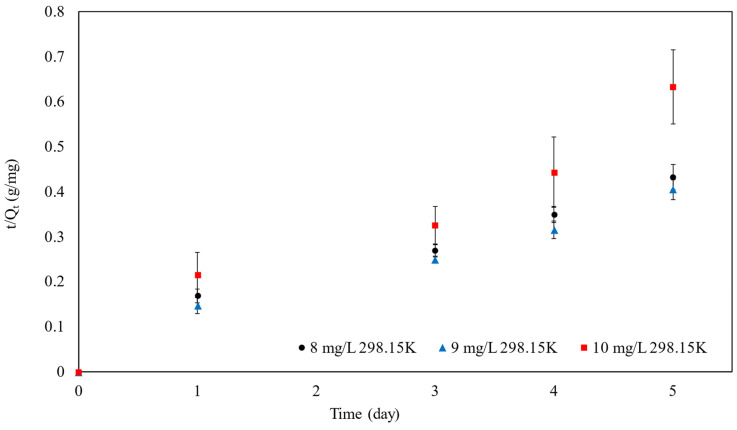
The pseudo-second-order kinetic model of SPION/M8C beads adsorbed MB.

Based on the loading amount over time, kinetic models were evaluated, as shown in Table 4 and Figure 11.

The R^2^ values obtained from the linear plot for pseudo-second-order kinetics, corresponding to MB concentrations of 8, 9, and 10 mg/L, were extracted from both Table 4 and Figure 11, revealing values of 0.96, 0.97, and 0.95, respectively. Additionally, it was noted that the Q_e_ and χ^2^ values derived from both pseudo-first-order and pseudo-second-order nonlinear models remained consistent. However, the adsorption kinetic model is best fitted as a pseudo-second-order linear model.

According to the pseudo-second-order kinetic model, it was inferred that MB molecules undergo chemisorption onto the surface of particles through the exchange of electrons, implying the involvement of valence forces between MB and SPION/M8C beads. Notably, this model suggests that the adsorption rate is influenced by concentration, with a quicker adsorption rate observed at lower MB concentrations compared to higher ones. This trend is supported by the observed reduction in the values of the rate constant (k_2_) as the concentration of MB increased, indicating a slower adsorption process at higher concentrations.

**Figure 12:**
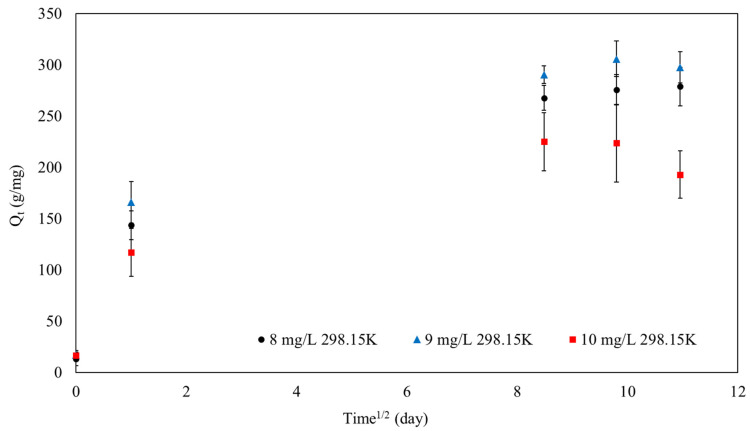
The IPD model of SPION/M8C beads adsorbed MB.

Additionally, the intraparticle diffusion kinetic model (IPD model) was also evaluated, as shown in Figure 12, to investigate the adsorption mechanism of MB onto SPION/M8C beads, particularly if the film diffusion process is one of the adsorption processes.

As demonstrated in Figure 12, the adsorption capabilities of MB onto SPION/M8C beads may be fitted via intraparticle diffusion. Weber’s intraparticle diffusion model may be used to compute the intraparticle diffusion rate. In the case when I = 0, intraparticle diffusion is the adsorption process. Table 5 displays the computed values of k_IPD_ and I.

Table 5 observes R^2^ values of 0.87, 0.85, and 0.80 for MB concentrations of 8 mg/L, 9 mg/L, and 10 mg/L, based on a linear plot of pseudo-second-order kinetics. These results show that the pseudo-second-order model fitted the experimental data quite well, especially at lower MB concentrations.

However, the analysis of the IPD model indicated that the intercept (I) did not equal zero. The difference proved that IPD had an important role in the adsorption process. The non-zero intercept suggested that the adsorption mechanism includes film and intraparticle diffusion [51,59].

**Figure 13:**
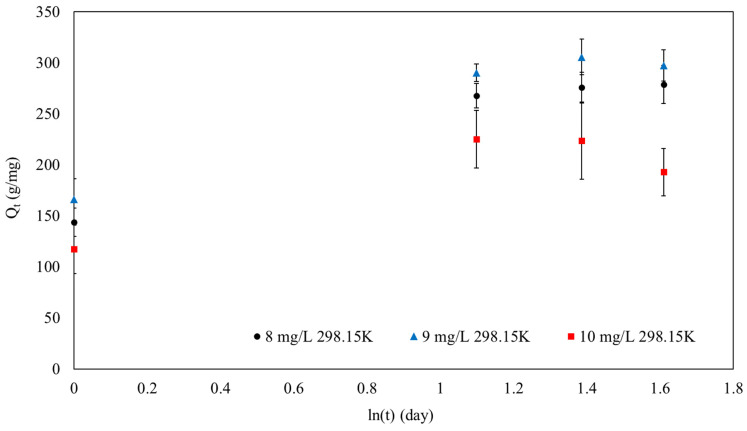
The Elovich model of SPION/M8C beads adsorbed MB.

Additionally, the Elovich kinetic model was also evaluated, as shown in Figure 13 and Table 6.

Figure 13 and Table 6 exhibit substantial R^2^ values from the Elovich model, indicating energy heterogeneity at the adsorbent surface. The R^2^ values of 0.95, 0.93, and 0.71 for MB concentrations of 8 mg/L, 9 mg/L, and 10 mg/L, respectively, confirm a strong fit for the model, particularly at lower concentrations.

Understanding the efficiency of the adsorption process relies heavily on the initial sorption rates, represented by the α values. At MB concentrations of 8, 9, and 10 mg/L, the initial sorption rates were determined to be 479.8, 616.74, and 514.30 mg/g·min per day. These data illustrated that the adsorbent had a high initial capacity for MB absorption.

As the concentration of MB rose from 8 mg/L to 10 mg/L, the β values, which represented the activation energy for chemisorption, fluctuated between 0.01 mg/g and 0.02 mg/g. The range of β values provided the energy required for chemisorption and suggested that the adsorption process was regulated by the concentration of MB. The range in these values emphasized the adsorption mechanism’s complexity and the importance of surface energy heterogeneity in the process.

### 3.8. Desorption

Even though the release of MB occurred at a constant temperature and a pH of 3.85, the release percentage of MB varied depending on initial loading conditions, such as temperature and starting MB concentration, as demonstrated in Table 7.

**Figure 14:**
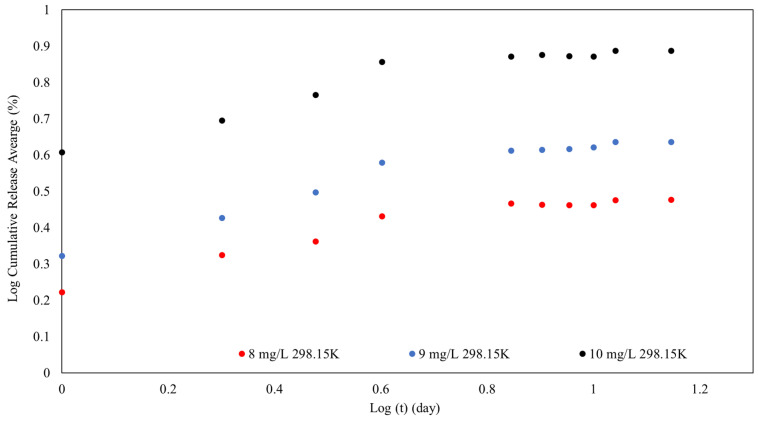
The releasing kinetics after 13 days of MB on SPION/M8C beads.

The release percentage of MB rose with increasing initial MB loading concentration. While the maximum release percentage was recorded at an initial concentration of 10 mg/L of MB, the trend indicated that increasing the initial MB concentration typically resulted in a greater release percentage. Additionally, the desorption kinetics were examined to investigate the desorption process, as shown in Figure 14.

Figure 14 expresses the progression from an initial fast-release phase to a more continuous and regulated release of MB from SPION/M8C beads. This found that the desorption process was controlled by variables such as the initial MB concentration.

The presence of SPIONs and polymers such as CS, PEG, PVA, and PVP could be responsible for the release profile seen in the graph. SPIONs may increase release rates in the presence of external magnetic fields, whereas polymers create a network that regulates diffusion and release kinetics.

**Figure 15:**
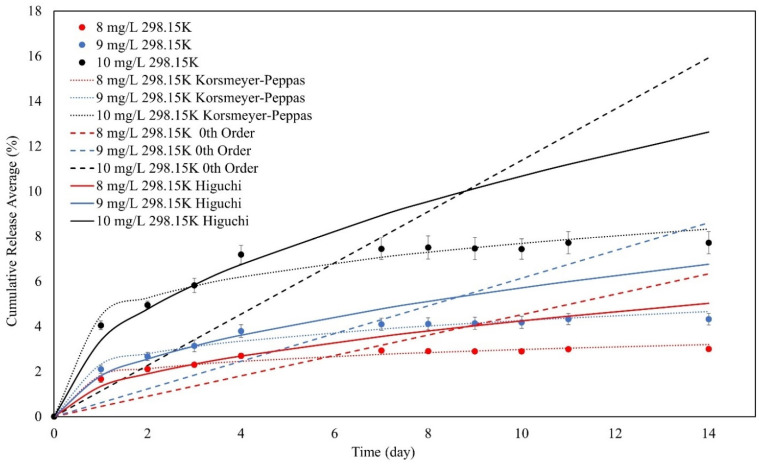
The Korsmeyer–Peppas, zeroth order, and Higuchi kinetic models for the desorption of SPION/M8C beads for MB.

Moreover, the best-fitting kinetic models were determined by combining the cumulative release percentage, the desorption kinetics models, and the χ^2^ values, according to Figure 15.

As shown in Figure 15, some of the models can fit the experimental data, and some cannot. To determine the most-fitted model and determine the desorption process, several parameters must be calculated, as shown in Table 8.

Based on the data in Table 8, the Korsmeyer–Peppas models provide release exponents (n values) that are less than 0.45, demonstrating a quasi-Fickian release mechanism. The quasi-Fickian release refers to a diffusion-controlled process in which the material is released from the matrix by a mix of diffusion and other processes. These variables may include polymer matrix relaxation, bead swelling, or other non-Fickian transport processes [60]. Furthermore, the release kinetics exhibit time-dependent behavior, as indicated by the values of n_KP_. This shows that the release rate changes over time, implying a dynamic interaction between the SPION/M8 beads and the released MB molecules.

All three of the models’ χ^2^ values were less than 1, but the zeroth order had the biggest, suggesting that the models did not fairly represent the experimental data. The KP’s χ^2^ value was the lowest. As a result, the KP model suited the desorption in this experiment the best.

## 4. Conclusions

In conclusion, the beads developed through the combination of polymers M8C, comprising CS, PEG, PVA, and PVP, encapsulating SPIONs represent a novel and cost-effective technique utilizing adsorption. This innovative approach offers antibacterial properties and ensures safety, presenting a promising solution to environmental challenges.

The synthesized SPIONs exhibited a diameter range of 19.03 ± 4.25 nm and a saturation magnetization value of approximately 51 emu/g. Moreover, the SPION/M8C beads displayed a total pore volume and pore diameter for adsorption peaking at 0.036 cm^3^/g and 9.822 Å, respectively. This was accompanied by the observation of a type IV isotherm, indicating adsorbents with wide pore size distributions.

Furthermore, the average SR of the beads exhibited a reduction from 12.78 ± 4.48 in cycle I to 8.24 ± 3.14 in cycle II, indicating a shift in the swelling behavior of the beads over successive cycles. Depending on the initial concentrations of MB, which were 8, 9, and 10 mg/L, the Q_t_ reached values of 278.68 ± 18.65, 297.48 ± 15.34, and 192.82 ± 23.18 mg/g, respectively. The %LC was recorded as 27.87 ± 1.87%, 29.75 ± 1.53%, and 19.28 ± 2.32%, while the %EE values were 29.42 ± 2.19%, 25.98 ± 1.29%, and 15.39 ± 1.83%, respectively. The pseudo-second-order kinetic model suggested that MB would chemisorb to the surface of the particles through the transfer of electrons between SPION/M8C beads and MB.

The cumulative release percentage of MB ranged from 3.00 ± 0.10% to 7.72 ± 0.50% when released at pH 3.85 over a duration of 13 days. Notably, the KP model was found to accurately represent the desorption kinetics observed in this experiment for the release of MB from SPION/M8C beads.

## Figures and Tables

**Table 1:** FTIR spectrum of M8C, SPIONs, and SPION/M8C beads.

Materials	Wavelength (cm^−1^)	Functional Group	References
M8C	2886	Asymmetric C–H stretching vibration	[54]
	1652	C=O stretching vibration of PVP, O–H bending mode of the –OH groups (due to the high amount of water), C=O stretching vibration of PEG, C=O stretching (Amide I) of CS + AA	[50]
	1107	C–O–C symmetric stretching of PEG free amino group –NH_2_ at the C2 position of glucosamine in CS + AA	[50]
SPIONs	3406	O–H stretching	[55]
	1625	Carbonyl C=O stretching band	[55]
	585	Vibration Fe–O	[56]
SPION/M8C beads	3430	–OH stretching of PVA, PVP, PEG –NH groups of CS + AA	[50]
	2923	Asymmetric C–H stretching vibration	[54]
	1640	C=O stretching vibration of PVP, O–H bending mode of the –OH groups (due to the high amount of water), C=O stretching vibration of PEG, C=O stretching (Amide I) of CS + AA	[50]
	1106	C–O–C symmetric stretching of PEG free amino group –NH_2_ at the C2 position of glucosamine in CS + AA	[50]
	580	Stretching of Fe–O Formation of FeO	[56]

**Table 2:** The BJH and BET analyses of SPION/M8C beads.

	Adsorption	Desorption
Surface area (m^2^/g)	25.89	16.71
Pore diameter (Å)	9.82	90.28
Pore volume (cm^3^/g)	0.036	0.034

**Table 3:** The Qt, %LC, and %EE of SPION/M8C beads adsorbed MB after 120 h.

Initial MB Concentration (mg/L)	Q_t_ (mg/g)	%LC	%EE
8	278.68 ± 18.65	27.87 ± 1.87	29.42 ± 2.19
9	297.48 ± 15.34	29.75 ± 1.53	25.98 ± 1.29
10	192.82 ± 23.18	19.28 ± 2.32	15.39 ± 1.83

**Table 4:** The pseudo-order kinetic model of SPION/M8C beads adsorbed MB after 120 h.

Model		Initial MB Concentration (mg/L)		
		8	9	10
Pseudo 1st order nonlinear	Q_e_	247.92	270.96	194.56
	k_1_	1.00	1.00	1.00
	χ^2^	52.35	49.15	40.00
Pseudo 2nd order nonlinear	Q_e_	247.92	270.96	194.56
	k_2_	171.82	171.82	171.82
	χ^2^	52.35	49.15	40.00
Pseudo 2nd order linear	Q_e_	12.59	13.48	8.94
	k_2_	0.17	0.18	0.39
	R^2^	0.96	0.97	0.95

**Table 5:** The IPD kinetic model of SPION/M8C beads adsorbed MB after 120 h.

	Initial MB Concentration (mg/L)		
	8	9	10
I	67.65	79.91	61.41
k_IPD_	21.18	22.37	15.47
R^2^	0.87	0.85	0.80

**Table 6:** The Elovich kinetic model of SPION/M8C beads adsorbed MB after 120 h.

	Initial MB Concentration (mg/L)		
	8	9	10
α	479.86	616.74	514.30
β	0.01	0.01	0.02
R^2^	0.95	0.93	0.71

**Table 7:** The percentage release average of MB from SPION/M8C beads after 13 days.

	Initial MB Concentration (mg/L)		
	8	9	10
% Release average	3.00	4.32	7.72
% Release standard deviation	0.10	0.26	0.50

**Table 8:** The Korsmeyer–Peppas, zeroth order, and Higuchi kinetic model calculations for the desorption of SPION/M8C beads for MB.

Initial MB Concentration (mg/L)	KP		0th Order	Higuchi	χ^2^		
	k_KP_	n_KP_	k_0_	k_H_	KP	0th Order	Higuchi
8	1.83	0.21	0.45	1.34	0.07	9.60	0.21
9	2.33	0.26	0.61	1.81	0.13	11.06	0.16
10	4.47	0.24	1.14	3.38	0.30	22.35	0.42

## Data Availability

All data that support the findings of this study are included within the article.
